# The Extracellular Matrix in Skin Inflammation and Infection

**DOI:** 10.3389/fcell.2021.682414

**Published:** 2021-07-06

**Authors:** Karin Pfisterer, Lisa E. Shaw, Dörte Symmank, Wolfgang Weninger

**Affiliations:** Department of Dermatology, Medical University of Vienna, Vienna, Austria

**Keywords:** extracellular matrix (ECM), cell migration, inflammation, infection, tissue homeostasis, skin, human

## Abstract

The extracellular matrix (ECM) is an integral component of all organs and plays a pivotal role in tissue homeostasis and repair. While the ECM was long thought to mostly have passive functions by providing physical stability to tissues, detailed characterization of its physical structure and biochemical properties have uncovered an unprecedented broad spectrum of functions. It is now clear that the ECM not only comprises the essential building block of tissues but also actively supports and maintains the dynamic interplay between tissue compartments as well as embedded resident and recruited inflammatory cells in response to pathologic stimuli. On the other hand, certain pathogens such as bacteria and viruses have evolved strategies that exploit ECM structures for infection of cells and tissues, and mutations in ECM proteins can give rise to a variety of genetic conditions. Here, we review the composition, structure and function of the ECM in cutaneous homeostasis, inflammatory skin diseases such as psoriasis and atopic dermatitis as well as infections as a paradigm for understanding its wider role in human health.

## Introduction

**FIGURE 1:**
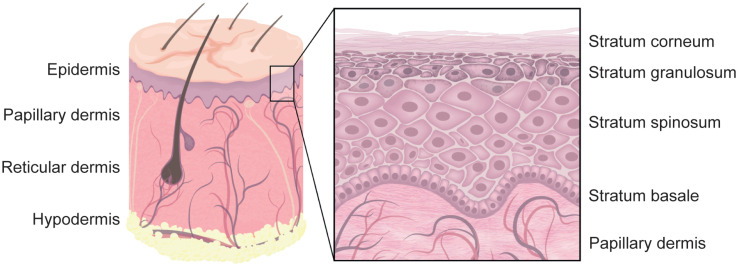
Human skin. The outermost layer of human skin is built by stratified epidermis. The bottom layer is called stratum basale and contains proliferating basal keratinocytes that are anchored to the basement membrane. While differentiating, they migrate from the stratum basale, through the stratum spinosum, stratum granulosum, and the stratum lucidum (not shown) to the stratum corneum. The vascularized dermis can further be separated into the upper papillary and the lower reticular dermis. Skin appendages, such as hair follicles, are embedded in the dermis and reach through the epidermis, whereas other appendages reside mostly in the dermis. The innermost skin layer is called hypodermis and is built by fat storing adipocytes. Note that not all skin appendages, and skin cells are shown.

As the body’s outermost surface, the skin must provide a tight barrier against a variety of physical and chemical noxae as well as pathogens, while at the same time facilitating communication with the environment. Schematically, human skin is arranged in three main layers. The epidermis is a stratified epithelium and is predominantly built by different layers of keratinocytes. Basal layer keratinocytes have a stem cell-like character, proliferate and then differentiate while moving from the *stratum basale* toward the outside of the skin. The underlying dermis is composed of fibroblasts, blood and lymphatic vessels, nerve fibers and extracellular matrix (ECM). Also embedded in the dermis are various immune cell subsets, skin appendages such as hair follicles, glands, and sensory organs. The hypodermis contains mainly fat cells and blood vessels (Figure 1). Similar to other organs, the ECM provides the necessary mechanical stability for the skin and, at least in part, enables its response to the environment. As such, the cutaneous ECM serves as a paradigm for our understanding of ECM composition, the relationship between tissue-resident immune cells and ECM as well as the adaptive response of the ECM toward external stimuli. Moreover, recent evidence suggests that the ECM plays a crucial part in preventing or facilitating cutaneous infections. In this review, we introduce general principles of ECM composition and remodeling. We will discuss how immune cells use the ECM for precise navigation through the extracellular space, a prerequisite for the proper function of immune responses. Finally, we will focus on ECM in the skin as a model of how inflammatory and infectious processes alter its biomechanical properties and how ECM components are usurped by infectious agents for breaching the body’s barrier tissues and infecting tissue-resident cells.

## Composition, Remodeling and Mechanical Characteristics of Skin ECM

The ECM is an evolutionarily conserved, highly flexible and dynamic scaffold that is constantly changed through controlled remodeling and restructuring processes. The different layers of the skin, i.e., epidermis and dermis, are separated by the basement membrane (BM), a complex mixture of ECM proteins and fibers that anchor the epithelium to the underlying tissue. Within the interstitial space of the dermis, cells such as fibroblasts, immune cells, and vascular cells are surrounded by and in tight interaction with the ECM which provides physical support to these cells. It also allows cell sensing of ever-changing conditions in the local milieu and facilitates cell-to-cell communication in order to adapt to such shifting microenvironments. Nevertheless, a deregulation in ECM composition can directly contribute to or induce pathological conditions and is a hallmark of the aging process (Bonnans et al., 2014). The ECM is further involved in relaying signals important for the recruitment of inflammatory cells in response to danger signals. Immune cells can use the ECM to migrate along biochemical gradients to sites of skin infection or tissue damage. However, components or mechanical properties of the matrix can also be exploited or actively changed. Pathogens, for instance, can use the ECM to invade into tissues and infect cells. Cancer cells can sense and alter mechanical properties such as stiffness of the ECM and exploit ECM stiffening to metastasize (Chang et al., 2017). In the following, we will highlight our understanding of ECM components (Table 1) and remodeling.

**TABLE 1:** Core extracellular matrix components in human skin.

ECM component	Localization within skin	Function
**Collagen**		
Collagen type I	Interstitial matrix, dermis	Most common collagen type in dermis, support, tensile strength, fibrillary
Collagen type III	Interstitial matrix, dermis	1/3 of fibrillary collagen in dermis, support, tensile strength, fibrillary; connected to Ehlers-Danlos syndrome
Collagen type IV	Basement membrane	Network-forming structure, highly flexible, provides strength, non-fibrillary
Collagen type V	Interstitial matrix, dermis	Minor collagen type in dermis, fibrillary; connected to Ehlers-Danlos syndrome
Collagen type VI	Papillary dermis and interstitial matrix	Links collagen IV to fibrillary collagen I and III in papillary dermis, binds decorin, biglycan, fibronectin, builds beaded microfibrils
Collagen type VII	Basement membrane, papillary dermis	Assembles into anchoring fibrils that anchor BM to papillary dermis; connected to EB
Collagen type XII	Interface between BM and papillary dermis	Fibril-associated, interacts with TSP-1, FACIT
Collagen type XIII	Keratinocyte surface	Transmembrane, ectodomain released by cleavage
Collagen type XIV	Interface between basement membrane and papillary matrix	Fibril-associated, interacts with TSP-1, FACIT
Collagen type XVII	Keratinocyte surface	Transmembrane, released by cleavage, attaches keratinocytes to basement membrane, regulates keratinocyte proliferation, maintains stem cells in hair follicles, MACIT
Collagen type XXIII	Keratinocyte surface	Transmembrane, released by cleavage, MACIT
**Elastic fibers**		
Elastin	Interstitial matrix, dermis, vessels	Elasticity and resilience; connected to Marfan syndrome and Cutis laxa
Fibrillin-1	Interstitial matrix, dermis	Predominant form, provides skin elasticity and flexibility, assists in BM anchorage to interstitial matrix, regulates TGF-β bioavailability, mechanosensor
Fibrillin-2	Interstitial matrix, dermis	Present during development and wound healing, similar functions as fibrillin-1
**Glycoproteins and matricellular proteins**		
Laminin (332, 311, 511)	Epidermal basement membrane	Stabilize epidermal adhesion and mediate epidermal-dermal communication, can interact with anchoring fibrils and collagen XVII, can polymerize into network
Fibronectin	Interstitial matrix, dermis	Interacts with collagen, provides adhesion cues for cells
Nidogen/entactin	Basement membrane	Colocalizes with laminin, important in embryonic development, bridging molecule, helps maintain epidermal integrity
TSP-1	Basement membrane, papillary dermis	Wound healing, stimulates cell proliferation, tissue homeostasis, stimulates ECM synthesis
Periostin	Papillary dermis	Wound healing, stimulates cell proliferation, involved in collagen deposition and scar formation
Tenascin C	Interstitial matrix, dermis, wound edges	Low expression, upregulated in wound healing, stimulates cell proliferation
**Proteoglycans**		
Biglycan	Interstitial matrix, dermis	Collagen-interacting protein, highly abundant in papillary dermis, regulates collagen fibril formation
Decorin	Interstitial matrix, dermis	Collagen-interacting protein, highly abundant in papillary dermis, regulates collagen fibril formation, stabilizes tissue
Perlecan	Basement membrane	Aids laminin-collagen IV linkage, signals via integrin binding and supports keratinocyte proliferation
Versican	Reticular dermis	Large chondroitin sulfate PG, promotes stability via interaction with elastic fibers and matricellular proteins, stimulates fibroblast proliferation
Fibromodulin	Interstitial matrix, dermis	Function uncertain
Lumican	Interstitial matrix, dermis	Function uncertain, involved in collagen fibrillogenesis
Agrin	Basement membrane	Not fully understood
**GAG**		
Heparan sulfate	Basement membrane	Involved in Wnt signaling, development, angiogenesis, metastasis, attachment site for viruses
Hyaluronan/hyaluronic acid	Interstitial matrix, dermis	Tissue repair, regulates tissue hydrodynamics by water binding, interacts with CD44
Dermatan sulfate	Interstitial matrix, dermis	Involved in wound repair, infection, fibrosis, carcinogenesis

*The list shown is not comprehensive. Further details on ECM proteins and associated diseases have been summarized in Uitto et al. (1989), Bosman and Stamenkovic (2003), Mouw et al. (2014), Fidler et al. (2018), Bhattacharjee et al. (2019), Nystrom and Bruckner-Tuderman (2019). PG, proteoglycan; GAG, galactosaminoglycan/glycosaminoglycan; HS, heparan sulfate; BM, basement membrane; TSP-1, thrombospondin 1; FACIT, fibril-associated collagens with interrupted triple helices; MACIT, membrane-associated collagens with interrupted triple helices; EB, epidermolysis bullosa, a severe skin disease that manifests in skin fragility and blistering (Bhattacharjee et al., 2019).*

## Composition of the ECM

### Collagens

**FIGURE 2:**
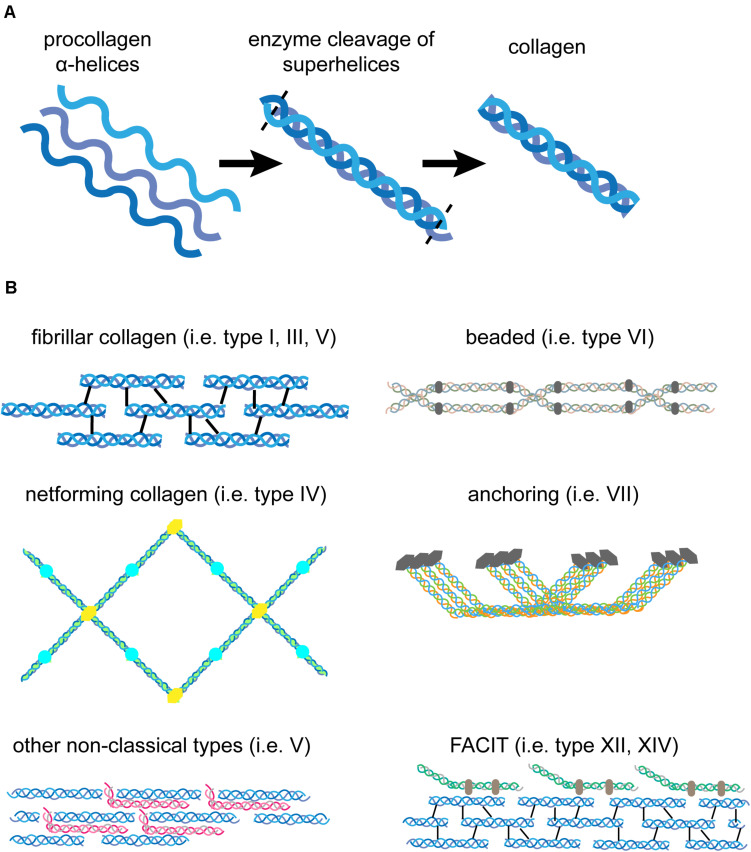
Structure of pro-collagen and assembled collagen. **(A)** Three left-handed α-helices (left) form a right-handed collagen superhelix (middle) within collagen-producing cells. Once secreted to the extracellular space, enzymes cleave off the N- and C-terminal parts to form fully functional collagen (right). **(B)** Different forms of collagen assemble into diverse structures. Fibrillar collagen molecules assemble into collagen fibrils that are stabilized by crosslinking (type I, III, V). The net-forming collagen type IV can build a meshwork that is highly flexible compared to rigid fibrils. Non-classical collagens, such as type V and FACIT interact with collagen fibrils to enhance stability. Beaded collagen (type VI) links collagen type IV to collagen type I and III and increases tissue elasticity. Collagen type VII assembles into fibrils that anchor the BM to the papillary dermis and maintain tissue integrity.

The major structural component of the matrix in human skin is collagen, a high-molecular weight protein that provides structure and tensile strength. Collagen molecules are evolutionary conserved in multicellular organisms and contain multiple Glycin (Gly)-X-Y repeats, in which X and Y are frequently proline and hydroxyprolin, but can be exchanged by any other amino acid as well (Mouw et al., 2014; Fidler et al., 2018). This amino acid sequence allows three collagen polypeptide chains (α-chains) to assemble into a stable triple helix at the rough endoplasmic reticulum of collagen producing cells (Fidler et al., 2018; Figure 2A). Folding initiates at the C-terminal domain. Right-handed pro-collagen superhelices are secreted into the extracellular space and the N- and C-terminal propeptides are cleaved off by enzymes resulting in fully functional collagen (Uitto et al., 1989; Mouw et al., 2014; Figure 2A).

**FIGURE 3:**
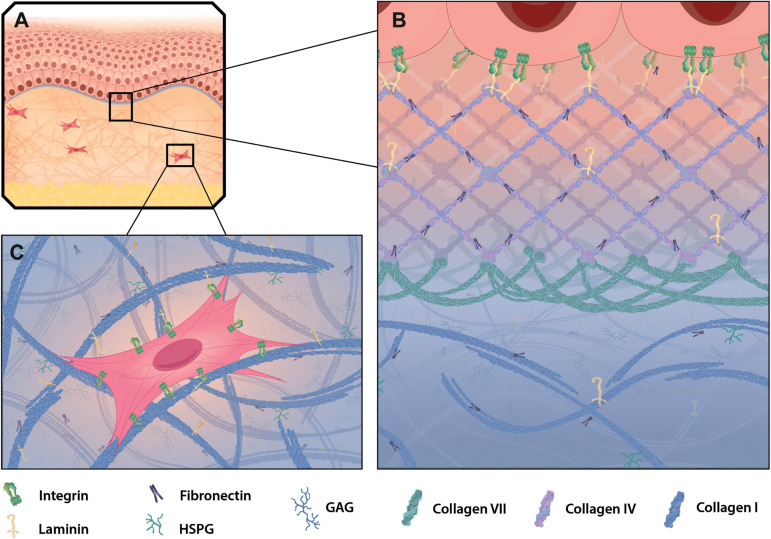
ECM in healthy mammalian skin. **(A)** Skin is composed of the epidermis (top part), dermis (mid part) and adipose cell-rich hypodermis (lower part) and resembles a highly accessible model tissue to study epithelia, connective tissue and extracellular matrix (ECM) structures specific for those layers. The BM (purple) connects the epidermis to the dermis, which is comprised of interstitial matrix. **(B)** The dominant structural component in the BM is collagen type IV, which builds a meshwork with interspersed laminin and fibronectin. Together they build the basis for keratinocyte attachment via integrins. Collagen type VII reaches into the papillary dermis and stabilizes the dermal-epidermal junction. **(C)** In the dermis collagen type I and fibronectin build a fibrillary structure that allows cell adhesion and migration through the matrix. Shown is a fibroblast attaching to collagen and fibronectin via integrins.

Collagens are produced and secreted primarily by fibroblasts, but other cells such as basal keratinocytes have also beenshown to express certain collagens, for example collagen type VII (Uitto et al., 1989; Nystrom and Bruckner-Tuderman, 2019). The fibrillary collagen types I, III, and V are the main collagen forms in the interstitial matrix of the dermis. Multiple processed fibrillary collagen molecules assemble and are further stabilized by intra- and intermolecular crosslinking to finally form collagen fibers of varying sizes (Mouw et al., 2014; Figure 2B). Only collagens type I, II, III, V, and XI self-assemble into fibrils, whereas the others need assistance from other matrix proteins or assemble into network-like structures that are typical in BMs (Bosman and Stamenkovic, 2003). Recent research has shown that the procollagen production and fibril formation is rhythmically regulated by the circadian day-night cycle (Chang et al., 2020). In human skin collagen type III is the most abundant version during prenatal development. However, after birth type I collagen becomes the dominant form in the dermis of adult skin (Uitto et al., 1989). Collagen type V is predominantly incorporated into existing collagen fibrils (Figure 2B). It inserts primarily between collagen type I strands and stabilizes them. Its abundance is low compared to the other two forms and the functional significance is still under investigation (Uitto et al., 1989; Bosman and Stamenkovic, 2003; Mouw et al., 2014). Collagen type IV is the major structural component of the BM (Uitto et al., 1989). It is structurally different from type I collagen, as it has intramolecular discontinuities of the sequential Gly-X-Y motifs, which allows more flexibility and the formation of a mesh-like network rather than a rigid, fibrillary structure characteristic for interstitial ECM (Uitto et al., 1989; Figures 2, 3). Collagen type VI is filamentous and builds beaded microfibrils (Figure 2B), which link collagen type IV to collagen type I and III in the papillary dermis. Collagen type VII assembles into anchoring fibrils, which are located in the interphase between the BM and the papillary dermis and are important for stabilization of the dermal-epidermal junction. As such, collagen type VII can maintain the BM-interstitial matrix integrity via interaction with type IV collagen (Uitto et al., 1989; Bosman and Stamenkovic, 2003; Figures 2B, 3). Interestingly, UV light has a direct effect on the matrix as it depletes type VII collagen, which ultimately leads to reduced deposition of type I collagen and skin aging (Watt and Fujiwara, 2011). Collagens type XII and XIV belong to the group of FACIT (fibril-associated collagens with interrupted triple helices). FACIT collagen does not form fibrils on its own, but associates with assembled collagen fibrils and further stabilizes them (Mouw et al., 2014; Figure 2B). Membrane-associated collagens with interrupted triple helices (MACIT) such as collagens type XIII, XVII and XXIII bind to the cell surface of keratinocytes and can be released by cleavage (Mouw et al., 2014). The MACIT collagen type XVII has been reported to maintain stem cells in hair follicles and its proteolysis by neutrophil elastases results in stem cell aging accompanied with hair follicle aging and alopecia (Matsumura et al., 2016). The role of other FACIT and MACIT molecules in skin is not yet well understood.

### Elastic Fibers

The two major components of elastic fibers are elastin and microfibrils. Fibrillins, such as fibrillin-1 and -2, are the predominant component of microfibrils that wrap around elastin, which builds the core of the elastic fiber. Elastin covalently links to collagen, fibrillin, fibulin and microfibril-associated glycoproteins and builds viscoelastic fibers, which out-compete other components of the ECM in regards to biological, chemical and thermal stability (Humphrey et al., 2014; Alfano et al., 2016). In contrast to collagen, which provides tensile strength, elastin is important for tissue elasticity and resilience to restrain stretching. Genetic deficiencies or structural abnormalities of collagen types or elastic fiber components can result in severe skin diseases, and have been reviewed recently (Fidler et al., 2018; Bhattacharjee et al., 2019; see also Table 1).

### Glycoproteins

Glycoproteins of the ECM have multiple functions; they stabilize collagen fibers during deposition, can add elasticity to guarantee flexibility or enhance rigidity. Fibronectin is the dominant glycoprotein in the interstitial matrix of the dermis and is mainly produced by fibroblasts and keratinocytes (Uitto et al., 1989; Bonnans et al., 2014). Laminin, like fibronectin, is a fibrous glycoprotein and a principal component of the sheet-like BM. The compact but flexible meshwork-like scaffold built by type IV collagen is a prerequisite for the interaction with and incorporation of BM molecules, such as laminin 5, perlecan and nidogen that link laminin to collagen type IV and heparan sulfate proteoglycans (HSPG) discussed later (Bosman and Stamenkovic, 2003; Bonnans et al., 2014). Tissue-resident cells are constantly interacting with the ECM and are reliant on provided anchor points for migration, proliferation and differentiation (Bonnans et al., 2014; Figure 3). Glycoproteins, such as fibronectin and laminin, provide those anchor points by acting as ligands for cell surface receptors. They bridge cellular integrins and collagen fibers, allowing efficient cell attachment to the matrix and also cell migration through tissues. Basal keratinocytes for instance preferentially bind to type IV collagen in the BM and this interaction is dependent on laminin 5 (Bosman and Stamenkovic, 2003; Watt and Fujiwara, 2011).

### PG/GAGs

Another central component of the core ECM are proteoglycans (PG), which are interspersed between and stick to collagen fibers in the ECM. Prominent PGs in the skin are biglycan, decorin, versican, fibromodulin, and lumican in the interstitial matrix of the dermis, and perlecan and agrin in the BM (Table 1). PGs are heavily glycosylated and decorated with glycosaminoglycan (GAG) side chains. These mostly high-molecular weight PG/GAG polysaccharides sequester water molecules and store ions and are therefore essential regulators of hydration and ion homeostasis in the skin. The PGs versican and decorin can bind the GAGs hyaluronan and dermatan sulfate, which are involved in wound repair, infection clearance and skin hydration (Bonnans et al., 2014; Mouw et al., 2014). HSPG with its core protein perlecan is a major PG/GAG in the BM and can interact with collagens (mainly type IV) and glycoproteins (fibronectin, laminin) to assemble and stabilize the ECM (Andriessen et al., 1997). PGs either localize to the intercellular space or to cell surfaces, such as HSPG or syndecan (Andriessen et al., 1997). PG/GAGs can interact with and sequester matrix-associated proteins, such as growth factors, signaling molecules and chemokines and are involved in the creation of matrix-dependent growth factor gradients (Hynes, 2009; Bonnans et al., 2014; Dyer, 2020). These signaling gradients are essential to developmental processes and also immune cell recruitment in infection and inflammation (Hynes, 2009). CXCL4 for instance is secreted by macrophages at the site of inflammation, and has a high affinity for HSPG present in the glycocalyx of the endothelial surface (Dyer, 2020). This localized chemokine presentation is crucial for controlling leukocyte recruitment into inflamed tissue (Dyer, 2020).

### Matricellular and Matrix-Associated Proteins

Matricellular proteins and matrix-associated proteins primarily have regulatory roles on either matrix fibers, cells embedded within the matrix or both.

Matricellular proteins are a defined group of dynamic regulatory proteins that possess binding sites for matrix molecules and cell surface receptors (Nystrom and Bruckner-Tuderman, 2019). These dynamic regulatory proteins are not structurally related, but share key characteristics such as secretion by certain cells, interaction with certain ECM fibers, or certain functional roles during development and wound healing (Nystrom and Bruckner-Tuderman, 2019). In skin, thrombospondin (TSP-1), periostin and tenascin C are of particular importance and are discussed in detail later. The matrix associates with a variety of ECM regulators, ECM-affiliated proteins and secreted signaling molecules, such as matrix modifying proteases and their inhibitors, cell growth factors and cytokines/chemokines (Hynes, 2009; Bonnans et al., 2014). Interactions with matrix fibers can limit the diffusion of these factors, build concentration hotspots of associated molecules by regulated binding and release, and enable ligand maturation of ECM-stored molecules.

In general, matrix-associated proteins act in two ways – either in their bound form or in a released soluble version, and both states can emerge interchangeably. The ECM can sequester, locally release and activate growth factors (EGF, FGF, VEGF) and signaling molecules (WNT, TGF-β; Alfano et al., 2016; Hynes, 2009). TGF-β for instance depends on a tight interaction with matrix molecules for its activation by mechanical forces exerted on the matrix (Lu et al., 2011). The latent TGF-β binding protein (LAP), which keeps TGF-β in an inactive conformation, is anchored to ECM proteins, like fibronectins. Mechanical strain can expose LAP-associated TGF-β and make it freely available for proteolytic modifications (Hynes, 2009). Many glycoproteins act as growth factor reservoirs and release matrix-bound growth factors after controlled proteolysis. Fibronectin binds VEGF and this interaction has been shown to be beneficial for chronic wound healing. Provision of artificially generated fibronectin/VEGF residues on an engineered matrix allowed simultaneous binding to cellular integrins and VEGF receptors on cells and strongly enhanced cell proliferation, migration and ameliorated wound healing in a diabetic mouse model (Martino et al., 2011). Intrinsic domains within the ECM can also act as ligands for growth factor receptors (Hynes, 2009). Several ECM molecules, such as the glycoproteins laminin, TSP-1 or tenascin, bear multiple repetitive domains that are homologous to EGF and can stimulate cell growth directly as solid-phase ligands or after release by proteolysis (Engel, 1989).

## ECM Remodeling – Matrix Deposition and Turnover

The ECM is a highly dynamic structure that is constantly remodeled to maintain tissue homeostasis. To be able to adapt to a changing environment the ECM is remodeled via *de novo* synthesis as well as proteolytic degradation that must be in equilibrium to maintain healthy tissues (Humphrey et al., 2014).

Fibroblasts and keratinocytes are major drivers of ECM remodeling in adult skin as they synthesize many ECM proteins and secrete matrix degrading enzymes (Kisling et al., 2019). Fibroblasts are highly abundant in the dermis, less homogeneous than previously assumed and have distinct matrix secretion profiles (Ghetti et al., 2018). Single-cell RNA sequencing of human skin samples identified six transcriptionally distinct fibroblast clusters (Vorstandlechner et al., 2020). Interestingly, a DPP4-expressing fibroblast type produces higher amounts of the ECM proteins type I collagen and fibronectin compared with DPP4-negative fibroblasts (Vorstandlechner et al., 2020). Fibroblasts also synthesize a great variety of matrix degrading enzymes and their regulators, such as matrix metalloproteases (MMPs), tissue inhibitors of MMPs (TIMPs), serine proteases (i.e., plasmins), granzymes, elastases and cathepsins that hydrolyze collagen, elastin, glycoproteins and PG/GAGs (Bonnans et al., 2014; Bhattacharjee et al., 2019). Further, immune cells secrete a plethora of cytokines and growth factors that can induce ECM synthesis or degradation (IL-1, IL-4, IL-6, IL-13, TGF-β, TNF). TGF-β plays a key role in controlling both processes (Liguori et al., 2011; Bhattacharjee et al., 2019). Immune cells have also been shown to be involved in ECM generation and deposition. Migrating macrophages deliver ECM components and control timely deposition of type IV collagen at the *de novo* formed BM in drosophila embryos during development (Matsubayashi et al., 2017). This mechanism could be conserved among species and potentially be important in malignant diseases displaying reactivation of developmental processes. Indeed, in humans, tumor-associated macrophages have the potential to express matrix molecules, such as PGs, fibronectin and collagen type I, V, and VI (Liguori et al., 2011). However, further studies will be needed to explore these processes in human embryogenesis, skin development and tissue malignancies.

### Wound Healing and the Immune System

Type-2 immune responses via interleukin-4 receptor α signaling have been shown to play a key role in skin repair after mechanical injury. IL-4 secretion by innate lymphoid cells and signaling in macrophages at the injury site controls the architecture and cross-linking of collagen fibrils as well as the diameter and strength of these fibrils (Knipper Johanna et al., 2015; Boothby et al., 2020). Collagen deposition and its structural regulation are particularly important in wound healing processes (Boothby et al., 2020). The earliest phase after skin injury is inflammation, followed by deposition of new connective tissue, called granulation tissue, and keratinocytes migrate over this newly formed granulation tissue. Fibronectin plays a special role by providing a provisional matrix for the formation of granulation tissue (Uitto et al., 1989). Fibroblasts are activated and differentiate into myofibroblasts with elevated alpha smooth muscle actin (α-SMA) expression, thereby increasing mechanical tension – an important mechanical step for rapid wound closure. Further, myofibroblasts synthesize ECM proteins in a TGF-β regulated manner (Watt and Fujiwara, 2011). TGF-β also induces periostin expression in fibroblasts and basal keratinocytes during wound repair. Periostin, which is predominantly expressed in collagen-rich tissues that have to withhold mechanical stresses, is upregulated in skin granulation tissue with protein levels peaking after 7 days and declining to normal levels thereafter (Norris et al., 2007; Zhou et al., 2010). Lack of periostin in mice increases collagen stiffness and decreases elasticity compared to wild-type mice (Norris et al., 2007). Interestingly, periostin is only found upon severe trauma and is not upregulated in the dermis after incisional wounding (Zhou et al., 2010). In adult skin the ratio of collagen type I:III is approximately 5:1, but in wound healing this ratio can shift and result in aberrant scar formation like keloids (Uitto et al., 1989). Despite clear differences in keloids and hypertrophic scars, both illustrate elevated periostin expression. However, hypertrophic scars contain thick collagen bundles with high levels of periostin surrounding the cells, whereas keloid scars lack cells with periostin around them (Zhou et al., 2010).

Transforming growth factor-β can also inhibit ECM degradation by downregulating matrix degrading enzymes such as MMP-1 and by upregulating TIMPs (Watt and Fujiwara, 2011). Matrix proteases dynamically remodel the ECM and this turnover results in both matrix remodeling and restructuring, and in matrix signaling through the release of matrix-associated proteins by cleavage. MMPs are Ca^2+^ dependent, Zn^2+^ containing endopeptidases that cleave intact collagen fibers and play a dominant role in ECM degradation and release of cytokines and chemokines. A plethora of MMPs have been described in humans and have been reviewed extensively (Lu et al., 2011; Bonnans et al., 2014; Kisling et al., 2019). In human skin MMP-1 is the major proteinase that degrades fibrillar collagen type I and III. The resulting collagen fragments can be further cleaved by MMP-3 and MMP-9 with an overall preference for collagen fibrils that have low amounts of crosslinking (Quan and Fisher, 2015). This leads to predominant degradation of loose collagen fibers and accumulation of highly crosslinked stiff collagen bundles during aging and in photodamaged skin (Quan and Fisher, 2015; Shao et al., 2019) which may contribute to enhanced tumor progression due to tissue stiffening, as has been reported for lysyl oxidase (LOX) (Levental et al., 2009).

## Changing the ECM Architecture by Posttranslational Modifications

Posttranslational modifications add another level of complexity and regulatory opportunity to the already diverse tissue- and disease-specific matrix (Fidler et al., 2018). For instance, collagen fibers can be crosslinked resulting in modified ECM architecture and mechanical properties. Inter- and intramolecular crosslinking reactions are catalyzed by enzymatic crosslinkers, such as LOX and transglutaminase, whereas glycation with reducing sugar residues represents a non-enzymatic process (Roy et al., 2010).

### Enzymatic Crosslinking

The enzyme LOX is one of the most prominent regulators of ECM topography. Together with lox-like enzymes (LOXL1-5) and lysyl hydroxylases these enzymes determine the extent of inter- and intramolecular crosslinking in the ECM and results in stiffening of the matrix (Cai et al., 2017; Rodriguez-Pascual and Rosell-Garcia, 2018; Vallet and Ricard-Blum, 2019).

Lysyl oxidase catalyzes the conversion of lysine and hydroxylysine residues into highly reactive aldehyde groups. Thereafter, these reactive groups spontaneously condense with other oxidized groups and form aldimine crosslinks (Schiff base) with amino groups from neighboring ECM components (Wolf et al., 2009; Grau-Bové et al., 2015). This copper-dependent process allows covalent crosslinking between multiple nearby collagen fibers, creating thick collagen bundles. It also allows crosslinks of collagen with elastin, and results in overall stiffening of the ECM (Lu et al., 2011).

The enzyme transglutaminase catalyzes a reaction between glutamine and lysine residues on adjacent ECM proteins creating covalent amide bonds and thereby alters ECM microstructures (Orban et al., 2004; Tse et al., 2018).

In human skin stromal cells generate LOX, and a recently defined fibroblast subset has been suggested to be the main LOX producer (Vorstandlechner et al., 2020). Collagen is not distributed and organized evenly throughout the dermis: it is rather loosely organized in the papillary dermis and densely packed and highly crosslinked in the reticular dermis (Watt and Fujiwara, 2011). This could be the result of a distinct distribution of fibroblast subsets within the dermis resulting in hotspots of LOX concentration. Further, LOX expression is regulated by hypoxia-inducible factors (HIFs) that are upregulated in hypoxic environments and conditions. HIFs are induced by inflammatory hypoxia, infections causing immune responses, or local hypoxia due to more O_2_ consumption (Deng et al., 2016). Connections to inflammatory skin diseases and infections will be discussed later. As mentioned before, aging and UV light can have an effect on skin elasticity and stiffness. This is partly mediated via UV-induced upregulation of LOX in photodamaged skin and enhanced by selective degradation of loose collagen fibers that are not crosslinked (Quan and Fisher, 2015; Shao et al., 2019). Another enzyme in human skin that is able to crosslink adjacent collagen fibers and is involved in fibrosis is transglutaminase. The ECM protein periostin, which is involved in collagen fibrillogenesis, may function as a substrate of LOX or transglutaminase (Norris et al., 2007). However, more research is needed to fully clarify these interactions mechanistically. Increased LOX expression and activity has been linked to cancer development and metastasis formation in various tissues and high LOX protein levels correlate with poor clinical prognosis (Bonnans et al., 2014). To date little is known about the physiological relevance of LOX for maintaining human skin homeostasis.

### Non-enzymatic Glycation

Glycation reactions happen spontaneously throughout the human body and involve the formation of advanced glycation end products (AGEs) from glucose or its metabolites. The carbonyl group of reducing sugars reacts with the amine groups of proteins and forms protein-AGE adducts with many intermediate products. The reaction includes multiple steps such as the formation of a Schiff base, Amadori rearrangements, and oxidation reactions creating highly reactive functional groups (Bansode et al., 2020). The protein-AGE adducts can form further intermolecular crosslinks with neighboring proteins (protein-AGE-protein; Bailey, 2001; Dandia et al., 2019).

In particular collagen fibrils of the ECM are frequently subject to glycation as they are highly accessible to sugars, such as glucose. As a result, the structure of the collagen matrix is altered and stiffened through collagen fiber bundling (Mason et al., 2013; Pfisterer et al., 2020). This can affect the attachment of other ECM components and thereby change the overall matrix composition. Glycation has also been shown to lower the surface charge of collagen fibrils, potentially altering charge associated molecule attachment, such as water, ions or similar (Bansode et al., 2020). Certain diseases and aging can increase glycation processes in the human body. For instance, glycation reactions are increased in diabetic patients as a result of hyperglycemia and AGE production generally increases with age (Dandia et al., 2019). Tissue dysfunction in elderly individuals is often caused by glycation-induced cross-linking and causes tissue stiffening (Bailey, 2001). Changes in the ECM composition and architecture determine the mechanical characteristics, such as stiffness and deformability and influence inflammatory skin diseases accompanied by altered cell-matrix interaction and cell migration (Ringer et al., 2017).

## Cell-Matrix Interaction, Migration and Mechanical Forces

Changes in the geometrical orientation of fibers and their crosslinking can change the viscoelastic properties, reduce tissue flexibility through stiffening of the matrix and inhibit permeability by building tight barriers that block entry of cells or drugs (DuFort et al., 2011). The ECM can also provide structures to enhance cell motility and it has been shown that matrix stiffening directly correlates with cancer cell migration, metastasis formation and patient survival (Levental et al., 2009). The interaction between cells and the matrix is of a reciprocal nature and highly dynamic, as both the cells and the matrix change spatially and temporally (van Helvert et al., 2018).

### Cell-Matrix Interaction

Cells use transmembrane adhesion receptors (i.e., integrins) to attach and link their cytoskeleton to matrix components (i.e., fibronectin). Multimodular intracellular adhesion complexes such as focal adhesions (FA) or podosomes build the connections between adhesion receptors and the cytoskeleton (Rafiq et al., 2019; Michael and Parsons, 2020).

The intracellular parts of adhesion receptors are coupled to the actin cytoskeleton via multifaceted, specialized adhesion complexes that can sense and exert forces, called FA (Michael and Parsons, 2020). In 2D environments, integrins activate cell spreading on fibronectin or collagens resulting in the formation of FA at cell margins, whereas FA in 3D are highly dynamic, more in number and not restricted to the base of the cell (Adams et al., 1999; Yamada and Sixt, 2019). A recent study using a FRET tension sensor to measure loads on integrins showed that matrix adhesion and traction force generation is strongly influenced by the presence of integrins and ligand identity (Tan et al., 2020). FA are essential for ECM pulling and pushing by the cell, and plaque molecules such as paxillin, talin and vinculin may play a central part in transmitting high forces on single integrin molecules (van Helvert et al., 2018; Yamada and Sixt, 2019; Tan et al., 2020). Dynamic mechanical links between the ECM, adhesion complexes comprising integrins and adapter molecules and the F-actin cytoskeleton are called molecular clutches (Elosegui-Artola et al., 2016). Molecular clutches translate rearward moving F-actin flow into traction force toward the ECM (van Helvert et al., 2018; Yamada and Sixt, 2019). The ‘molecular clutch model’ involves repeated cycles of high force engagement and disengagement to the matrix via integrins/cadherins in FA and is involved in mechanosensing (Elosegui-Artola et al., 2016). The clutch model has recently been challenged using FRET-tension sensors, and this showed that there is less load on each sensor and for a long period of time, which is in contrast to high energy rapid engagement and disengagement. Force transmission could be rather realized through a network of dynamic actin crosslinkers, which may be energetically favorable (Tan et al., 2020). Vinculin and talin function as molecular clutch through direct but transient interaction with F-actin fibers to translate rearward moving F-actin flow into traction force toward the ECM (van Helvert et al., 2018; Yamada and Sixt, 2019). Podosomes, in contrast, are cellular multimodular matrix attachment complexes that are common in myeloid cells and build localized attachment foci, which are not limited to cell edges (Rafiq et al., 2019). Podosomes have an actin-rich core and an outer ring-like structure built by adhesion molecules, such as integrins, and plaque molecules, such as paxillin, talin and vinculin, that link integrins to the actin cytoskeleton (van den Dries et al., 2019). The serine/threonine kinase p21-Activated kinase 4 (PAK4) has been shown to localize to the ring-core interphase and is essential for podosome formation, dynamics and consequently macrophage migration (Foxall et al., 2019). A recent study showed that the formation of FA and podosomes is regulated via interactions between actomyosin networks and microtubules (Rafiq et al., 2019). KANK proteins bridge talin with the tips of microtubules. Uncoupling activates Rho/ROCK signaling and enhances myosin II filament assembly resulting in a preferred formation of focal adhesions over podosomes (Rafiq et al., 2019). Vinculin and talin also localize to adhesion rings on actin-rich membrane projections called invadopodia (Weaver, 2006). Invasive squamous cell carcinoma cells use these specialized matrix-degrading cell structures that are stabilized by podoplanin to remodel ECM structures in a LIM kinase-dependent pathway for tissue invasion (Scott et al., 2010; Martín-Villar et al., 2015).

Cells constantly need to sense changes in the properties of the ECM to allow them to adjust their behavior accordingly. Exploration of the matrix is often performed with actin-rich membrane protrusions, called filopodia. It has been shown that cell adhesion to ECM proteins, such as α5β1 integrin to fibronectin, but not adhesion to laminin or TSP-1, directly influences filopodia generation and stability by changing the PKC-dependent phosphorylation of fascin (Adams and Schwartz, 2000). Fascin bundles linear F-actin filaments in filopodia and is highly upregulated in migratory cancer cells, dendritic cells and Langerhans cells of the skin (Adams et al., 1999). In addition, fascin is also differentially phosphorylated in response to the stiffness of the ECM, suggesting that both, tight cell adhesion to fibronectin and interaction with stiff matrix, may influence the spatio-temporal distribution of fascin and thereby regulate filopodia function and activity (Pfisterer et al., 2020). Loss of matrix anchorage and its associated signaling can lead to the induction of a specialized form of programmed cell death, called anoikis. This can either be a result of matrix degradation or tension release within the matrix through wounding (Humphrey et al., 2014). This regulatory mechanism is often inactive in invasive tumor cells that metastasize and may play a role in hyperproliferative skin diseases, such as psoriasis, as well (Bonnans et al., 2014).

### ECM and Cell Migration

Cell migration through ECM is a finely tuned balance of adhesion and detachment from the matrix concomitant with continuous cell deformation to enable movement. If cells adhere too tightly to the matrix, movement is limited and cells will become static. In contrast, the lack of anchor points or mechanical resistance can result in uncontrolled slippage on the matrix, with the consequence of reduced or halted cell migration (Yamada and Sixt, 2019). Before the onset of movement, many migratory cells explore the ECM with filopodia or sheet-like membrane protrusions called lamellipodia at the cell front. This allows sensing of the microenvironment and direct adaptation to the ECM topography, stiffness and anchor possibilities (Pfisterer et al., 2020). Once the cell has successfully explored the ECM, filopodia are pulled in and cells mostly migrate in a filopodia-independent mode (Leithner et al., 2016).

**FIGURE 4:**
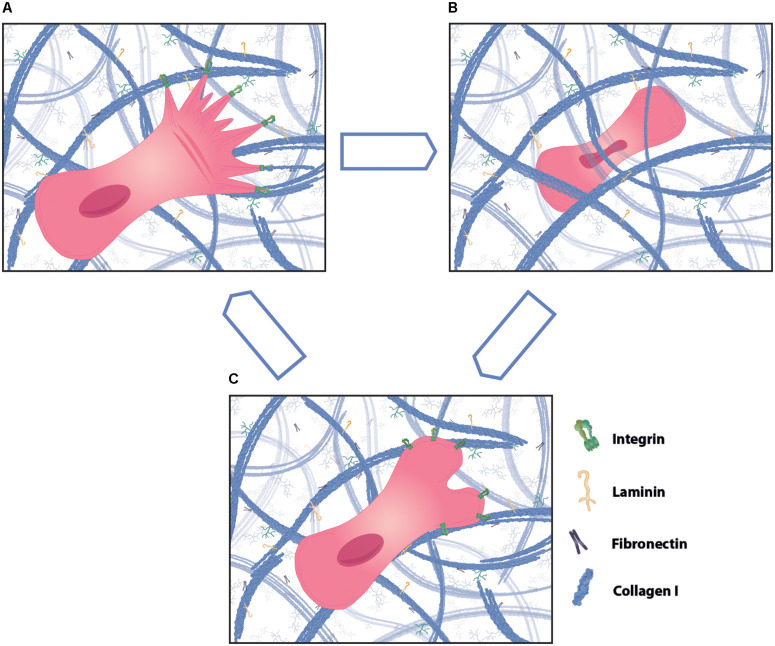
Different modes of cell migration in three-dimensional (3D) matrix. Cells can migrate through 3D matrix using different ways of motility, such as mesenchymal, amoeboid and lobopodial migration. **(A)** Mesenchymal migration is characterized by high adhesions and cell protrusions at the leading edge that align along collagen fibers. Cells that mainly use mesenchymal migration leave behind a tunnel in the ECM that can be utilized by following cells as tracks. **(B)** In contrast, amoeboid cell migration is less dependent on matrix adhesions. Cells show strong cell body and nucleus deformation during migration through tight spaces and generally choose the path of least resistance. **(C)** Lobopodial migration shows features of mesenchymal and amoeboid migration and typically has bleb protrusions at the migration front. Cells using one form of migration can change to using another form dependent on the environment.

Cell migration in 2D environments has been extensively studied and Michael Abercrombie postulated already in the 1970s the basic principles of 2D cell migration, consisting of repeated cycles of membrane protrusion at the leading edge (lamellipodia), adhesion, rearward movement of F-actin, and cell retraction at the rear of the cell (Abercrombie, 1980). F-actin polymerization at the front of the cell drives membrane protrusions, such as lamellipodia, filopodia, membrane blebs or invadopodia, and these processes are tightly regulated by Rho GTPases (Ridley, 2011; Lawson and Ridley, 2018). In contrast to flat 2D environments, cells use various migration modes in 3D, such as mesenchymal, amoeboid and lobopodial cell migration (reviewed in Yamada and Sixt, 2019 and illustrated in Figure 4). Leukocytes that patrol through the skin for homeostatic surveillance mainly use amoeboid migration, where they choose the path of least resistance, using low adhesion points and showcasing extensive cell deformation (Weninger et al., 2014; Yamada and Sixt, 2019). Stromal cells, such as fibroblasts, align along and attach to ECM tension fibers during mesenchymal migration and often leave behind tunnels due to MMP degradation at the front. Lobopodial migration shows features of both amoeboid and mesenchymal migration in addition to bleb-like protrusions at the front, which are generated via intracellular pressure (Yamada and Sixt, 2019). Cells can interconvert between these forms and the mode is dependent on substrate adhesion, actin protrusion, actomyosin contraction and of the physical, chemical and architectural properties of the matrix (Yamada and Sixt, 2019).

### Mechanotransduction and Forces

An important signaling mechanism is provided through reciprocal force generation between cells and the matrix. Loads on the ECM are sensed by cells and can result in enhanced Rho GTPase signaling and myosin contractility (Yamada and Sixt, 2019). In addition, cells within tissues are under constant mechanical tension, have to squeeze through tight spaces and thereby also exert pulling and pushing forces on the matrix or create tunnels through contractile forces in an otherwise dense matrix.

Physical parameters that influence cell migration are ECM stiffness, confinement and topology and the cell needs to adapt to these mechanical features and its remodeling (van Helvert et al., 2018; Yamada and Sixt, 2019). The density of collagen fibrils varies in tissues within the human body and determines its porosity (van Helvert et al., 2018). Porosity in combination with fiber alignment, matrix crosslinking and network tension define the physical properties of the ECM and can allow cell migration or prevent it. Small pore sizes for instance can be size selective or only allow cells to migrate through that can flexibly adjust to small spaces. The prerequisites of cell flexibility include active remodeling of the F-actin and microtubule cytoskeleton, and the deformation of the cell body and organelles. The nucleus is one of the most rigid organelles in eukaryotic cells that contains and protects the genetic information. Nuclear plasticity during cell migration is a prerequisite for efficient tissue penetration and migration through tight interstitial spaces. Fascin has been shown to regulate nuclear movement and flexibility by interacting with nesprin-2 at the outer nuclear envelope and thereby coupling F-actin to the nucleus (Jayo et al., 2016).

However, increasing confinement can quickly become a limiting factor, as seen during cellular transmigration through the BM. Nuclear rupture and content spillage can occur and cause DNA fragmentation. An exciting study using microfluidics to image nuclei during cell migration showed that after migration through pores sized smaller than 20 μm^2^, cells lost integrity of the nuclear membrane (Denais et al., 2016). However, cells developed rescue mechanisms that quickly restored nuclear content using the endosomal sorting complex required for transport III (ESCRTIII) machinery. Nuclear content was pulled in, followed by nuclear membrane and DNA damage repair and resulting in increased cell survival (Denais et al., 2016). Another solution for successful migration through ECM of restrictive physical properties is active ECM remodeling. Matrix stiffening can induce MMP secretion to break down ECM components at the leading edge, in podosomes and invadosomes (Chang et al., 2017). By utilising this, cells can create migration tracks behind them to form “superhighways” that can be used by trailing cells (Lu et al., 2011).

Cells and ECM form tissues and through dynamic reciprocal force transduction those tissues are dynamic entities. Changes in the physical properties of the ECM, such as pathological stiffening, are directly sensed by cells and have a direct impact on cell-interaction with the ECM and consequently cell migration (Paszek et al., 2005). Cells sense force loads on the ECM with the actin-talin-integrin-fibronectin clutch in a dynamic mechanism and force transmission has been predicted to follow a biphasic force/rigidity curve (Elosegui-Artola et al., 2016). Cell contraction results in intracellular forces that can be translated into forward movement. Non-muscle myosin II on F-actin fibers generates sufficient forces to actively retract the rear of the cell. Recently, it has been shown in a 3D ECM model that protrusion speed influences cell retraction by transduction of membrane tension in a positive feed-back loop (Hetmanski et al., 2019). A decrease of rear membrane tension as a result of polarized stiffness and migration, increases caveolae formation and this promotes RhoA activation and cell retraction (Hetmanski et al., 2019). In addition to sensing the stiffness, cells can also sense matrix geometry. The orientation and the alignment of ECM fibers can influence guided cell migration, the thickness of collagen fibers may have an impact on migration speed, and complex ECM topography and fiber nanotexture may provide anchor points for migrating cells (van Helvert et al., 2018). Exciting new research showed recently that adhesion-independent leukocyte migration is impossible on 2D surfaces due to the lack of substrate attachment. In contrast, locomotion through 3D ECM was similar between talin-deficient and wild-type cells and this was dependent on rough 3D topography to allow cells to use friction, and actin retrograde flow for non-adhesive forward movement (Bodor et al., 2020; Reversat et al., 2020).

Bi-directional reciprocity of forces between cells and the matrix can expose otherwise inaccessible cryptic peptide sequences on the ECM, open mechanosensitive ion channels, change receptor or ligand conformation to strengthen their interaction, and thereby alter cell-matrix interactions and biochemical signaling responses (Vogel and Sheetz, 2006; Wang et al., 2019). The interstitial matrix is a reservoir of signaling molecules. Concentration gradients of chemokines, growth factors, and morphogens regulate and direct cell migratory behavior. The ECM is also the largest source of freely available Ca^2+^ with a concentration of more than 1 mM (Gopal et al., 2020). As a secondary messenger, Ca^2+^ plays a dominant role in many cellular signaling pathways regulating immune cell activation, apoptosis, dynamic cytoskeletal rearrangements, cell adhesion, and migration. Besides sensing substrate rigidity through mechanosensitive adapter proteins, cells can also utilize mechanosensitive ion channels that are often located in close vicinity to FA (Gopal et al., 2020). High force load on the matrix can be translated via FA to actin stress fibers and ultimately result in opening of mechanosensitive Ca^2+^ channels. Direct pulling on single actin stress fibers using laser tweezers has also been shown to activate ion channels and result in localized increases in cytoplasmic Ca^2+^ concentrations due to high influx from extracellular stores (Kobayashi and Sokabe, 2010). Cell binding to and pulling on matrix-bound growth factors, cytokines or chemokines may enhance cell activation and also increase target cell specificity compared to soluble factors (Engel, 1989). Counterforces within the cell may be essential for activating signaling cascades (Chang et al., 2017). One explanation could be that increased cellular forces through interaction with rigid matrix and pulling on actin filaments may enhance cell signaling responses (Chang et al., 2017). Force sensing of the ECM environment can be seen in many cells and cells have been shown to migrate toward gradients of higher stiffness in a mechanism called durotaxis (Sunyer and Trepat, 2020). Changes of tissue integrity, ECM topology, rigidity, and composition can all modify cell migration and strongly affect tissue homeostasis.

## The Role of Skin Inflammation and Infection in ECM Remodeling

### Role of the ECM in Inflammatory Skin Diseases

**FIGURE 5:**
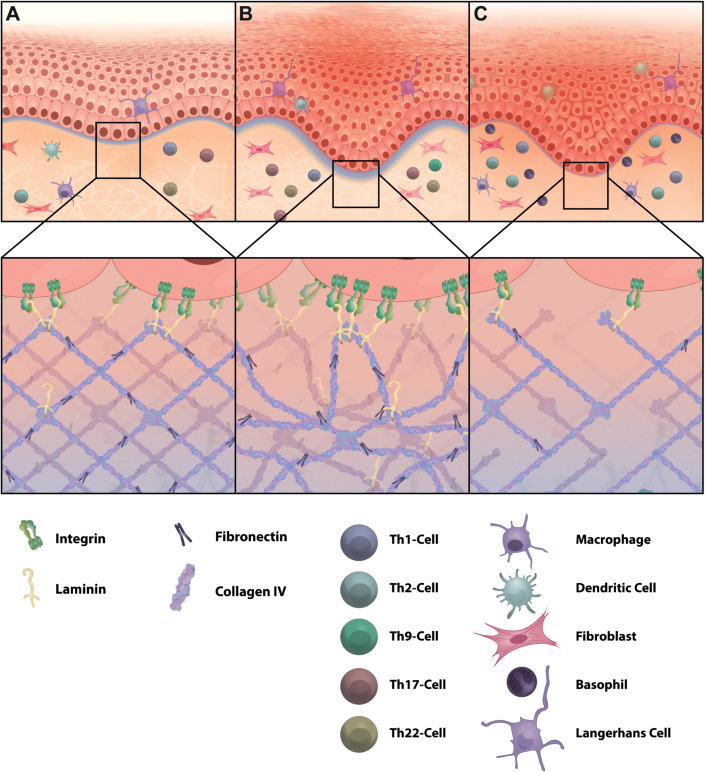
ECM changes in skin inflammation. Inflammatory skin diseases can alter the composition and structure of the ECM. **(A)** In healthy skin collagen type IV builds a network with interspersed laminin to form the BM. This provides attachment points for basal keratinocytes of the epidermis and forms a protective layer to control leukocyte navigation through adjusted gap sizes and limits entry of harmful invaders via incorporation of sticky PG/GAGs. **(B)** Psoriasis is defined by high infiltration of IL-17 and IL-22 producing T helper cells. The BM in psoriasis is highly unstructured and thicker compared to healthy skin and shows elevated levels of laminin and fibronectin. **(C)** In atopic dermatitis, which is a T helper 2-driven inflammatory skin disease, collagen type IV and fibronectin are decreased, resulting in a thinner BM. Increased hyaluronan and MMPs are further characteristic for atopic dermatitis (not illustrated here). Not all relevant immune cells are illustrated.

Extracellular matrix proteins are essential players in the regulation of tissue responses toward external and internal stimuli. If ECM remodeling is out of equilibrium, pathological conditions are exacerbated. Genetic deletions of specific ECM proteins can lead to tissue defects and can even be lethal in development (Bonnans et al., 2014). Due to the dynamic nature of the ECM, elevated remodeling rates may increase the risk of aberrant modifications, especially in tissue injury and inflammation (Figure 5).

Immune cells are major regulators in ECM synthesis and deposition, degradation and remodeling. Immune cells produce proteases (e.g., MMPs), cytokines (e.g., IL-1b, IFN-y), growth factors (e.g., TGF-β) and ECM components (e.g., fibronectin) that are involved in the remodeling of ECM components (Watt and Fujiwara, 2011; Bhattacharjee et al., 2019). In contrast, degradation of ECM proteins, such as hyaluronic acid, HSPG, and tenascin C can create fragments that induce inflammation and provide cues for immune cell recruitment (Adair-Kirk and Senior, 2008; Kurbet et al., 2016). Laminin fragments can act as danger associated molecular patterns (DAMPs) and modulate cytokine and MMP expression (Simon and Bromberg, 2017; Michopoulou et al., 2020). Collagen fragments increase IL-1β secretion in monocytes, whereas elastin peptides act as chemoattractant for immune cells (Adair-Kirk and Senior, 2008). Fibronectin splicing variants can activate toll like receptor (TLR) signaling, and cytokine and chemokine secretion in fibroblasts (Kelsh et al., 2014). Fibronectin has been associated with two major inflammatory skin diseases, psoriasis and atopic dermatitis (AD; Bhattacharjee et al., 2019).

Atopic dermatitis is a T helper 2 (Th2)-driven chronic inflammatory skin disease that also shows aberrant BM composition in affected skin (Figure 5). AD patients display reduced skin barrier function, which is caused by filaggrin mutations and reduced thickness of the BM with decreased amounts of collagen type IV and fibronectin (Bhattacharjee et al., 2019). Further, fibronectin redistributes to the cornified layer of uninvolved skin in AD patients, which allows bacterial colonization. *Staphylococcus aureus* (*S. aureus)*, the dominant pathogen on the skin of AD patients, has been shown to bind preferentially to fibronectin in the stratum corneum of AD and not psoriasis patients (Cho et al., 2001). *S. aureus* often causes chronic infections, results in increased immune cell infiltration to fight the pathogen and can cause tissue damage through proteolysis by bacterial elastases (Alfano et al., 2016). Chronic AD lesions are often characterized by eosinophil infiltration. Skin thickening in AD has been connected to secretion of leukotriene C4 by eosinophils, and fibroblasts respond with increased collagen synthesis and secretion (Oyoshi et al., 2012). Immune cell-induced MMP-dependent ECM degradation also plays a role in AD progression and AD patients have elevated serum levels of MMP-8 and MMP-9. Increased macrophage infiltration in chronic AD may also adversely affect ECM homeostasis by enhanced MMP activity (Bhattacharjee et al., 2019). In an attempt to investigate the link between pruritus, a common symptom in AD, and nerve fiber elongation in the dermis, upregulated MMP-8 has been shown to be involved (Tominaga et al., 2011). MMP-8 is induced by nerve growth factors secreted from keratinocytes and allows nerve fibers to penetrate into the ECM. The resulting increase in nerve fiber density in the interstitial matrix may explain the development and enhancement of pruritus, which is typical for AD lesions (Tominaga et al., 2011). AD is associated with IgE sensitization to various allergens and resulting allergic inflammatory reactions are often the cause and consequence of tissue remodeling. Allergic reactions may provoke histamine secretion by mast cells and this can trigger inflammatory reactions and itching sensation in AD patients as well. Histamine has also been shown to induce periostin-dependent collagen expression and deposition by fibroblasts via ERK1/2 signaling through the histamine receptor 1 and thereby regulates ECM remodeling (Yang et al., 2014). Periostin is highly expressed in the papillary dermis of embryonic skin and to a lesser extent in healthy adult skin. However, periostin is upregulated in wound healing processes and has also been reported to be highly abundant in AD skin (Zhou et al., 2010; Mishra et al., 2020). A recent study showed that periostin expression in keratinocytes is induced by thymic stromal lymphopoietin, calcipotriol and house dust mite, and is a potential inducer of allergic itch through engagement with αVβ3 integrin receptors on sensory neurons (Mishra et al., 2020).

In contrast to AD, periostin is reduced in psoriasis and the BM is thicker and unstructured, which further highlights the molecular and structural differences of these two skin diseases (Zhou et al., 2010; Bhattacharjee et al., 2019; Figure 5). Psoriasis is mainly driven by Th17/Th22 cell skin infiltration and cytokine secretion. High immune cell infiltration in psoriasis can also affect the structure and composition of the ECM. In addition to T cells and macrophages, neutrophils are recruited to wounded skin and are elevated in numbers in psoriatic skin lesions (Bhattacharjee et al., 2019; Boothby et al., 2020). Neutrophils can release elastase, which promotes elastic fiber degradation and MMP activation and could play a role in disease initiation by altering the matrix (Bonnans et al., 2014; Bhattacharjee et al., 2019). Recently, it has been reported that psoriatic non-lesional and lesional skin harbors high numbers of IL-9 producing Th9 cells in addition to Th17 cells. IL-9 abrogates keratinocyte migration by reducing actin stress fibers and actomyosin contractility, whereas IL-17A promotes MMP secretion in keratinocytes (Das et al., 2019). As such, both interleukins may act directly or indirectly on collagen composition and mechanical tension exerted on the matrix by keratinocytes, respectively. Psoriasis has been clinically linked to cardiovascular diseases, representing a major comorbidity factor. Huang et al. (2019) showed that cholesterol accumulates in tissues and cannot be cleared in an imiquimod-induced experimental psoriasis mouse model. Consistent with psoriasis in humans, Th17 cells are highly abundant in skin of imiquimod-treated mice. Increased IL-17 levels promoted collagen deposition, thickening of the collagen matrix and increased LOX-induced collagen crosslinking in the skin. As a result, relatively large high density lipoprotein (HDL) molecules get trapped in the high-density collagen structures and are unable to efficiently remove low density lipoprotein (LDL) from tissue and recirculate it to the plasma (Huang et al., 2019). The diminished lipoprotein trafficking may represent a mechanism leading to increased cardiovascular diseases in psoriasis patients and highlights the role of ECM topology and charge on molecule movement in the interstitium.

Certain medications that are commonly used to treat psoriasis and atopic dermatitis, can also induce ECM changes in the skin. Topical glucocorticoids have proven effective in decreasing inflammation and skin irritation and can restore homeostasis (Sarkar et al., 2017). However, they come at the expense of ECM integrity. Corticosteroids can modify connective tissues and result in dermal atrophy and striae formation (Uitto et al., 1989). These modifications are mainly evoked through inhibition of fibroblast proliferation, suppression of collagen synthesis by fibroblasts, and reduction in collagenase and MMP secretion. Betamethasone, which is regularly used to treat psoriasis, can also suppress ECM expression in fibroblasts and keratinocytes (Norsgaard et al., 2014). Though, combined therapy with the vitamin D derivate calcipotriol can restore collagen and hyaluronic acid levels and prevent dermal and epidermal thinning (Norsgaard et al., 2014). Ascorbic acid (vitamin C) also enhances collagen deposition by increasing collagen type I and III transcription in fibroblasts, and by stabilizing triple-helical collagen fibers for optimized secretion (Uitto et al., 1989).

### Role of the ECM in Infection

Components of the ECM are ubiquitously distributed throughout the human body and therefore attractive targets for pathogens for adhesion (Singh et al., 2012). Bacteria and viruses share similar strategies to guarantee successful infection of host cells in various tissues without being limited to specific tissue types. Infections often cause severe tissue damage and inflammation and induce remodeling processes to restore tissue homeostasis. As a consequence of tissue degradation, novel ECM binding sites for pathogens can be exposed and can potentially increase further bacterial or viral adhesion and infection (Singh et al., 2012).

Viruses can either spread directly from cell to cell using already established cell–cell interactions, activate the cell cytoskeleton to induce *de novo* cell–cell interactions, like nanotube bridges, or diffuse through the ECM from cell to cell (Sowinski et al., 2008; Zhong et al., 2013). Free diffusion through the ECM requires high virus stability, and efficient strategies for release from infected cells and infection of new cells. Attachment to sticky ECM components that surround cells can enrich virus particles close to the cell membrane for advanced infection.

### Pathogen Binding to ECM Components

Many bacteria and viruses utilize ECM proteins for adhesion and invasion and this simple yet sophisticated strategy seems to prove efficient (Singh et al., 2012; Table 2). *S. aureus* for instance binds the glycoproteins laminin, collagen, elastin and fibronectin, whereas many HPV subtypes use HSPGs for attachment (Bienkowska-Haba and Sapp, 2011; Singh et al., 2012). HPV-11 enters tissues through epithelial microlesions and binds via its capsid to laminin 5, which is secreted by migrating and basal keratinocytes during wound repair (Culp et al., 2006). Thereby, HPV elegantly exploits the interaction of α6-integrin with laminin 5 to invade into basal keratinocytes (Singh et al., 2012). Polyomaviruses have been shown to attach with their large T-antigen to laminin and collagen type IV at the BM (Singh et al., 2012). In contrast, Merkel cell polyomavirus, which is highly prevalent in the human population and the causative agent of Merkel cell carcinoma in 80% of patients, depends on sulfated polysaccharides, such as heparan sulfate (HS) for attachment before it engages sialic acid receptors on fibroblasts to invade (Matrosovich et al., 2013; Becker et al., 2019). Undersulfation of surface-bound GAGs, which are essential for initial cell attachment, reduced infection rates of Merkel cell polyomavirus in *in vitro* experiments (Becker et al., 2019). Various other viruses, such as herpes simplex (HSV) and HPV-types also depend on sulfated GAGs such as HS for initial binding (Stavolone and Lionetti, 2017; Hadigal et al., 2020). HPV-16 for instance uses HSPG to attach to the BM, to the interstitial matrix or to cell membranes prior to cell infection (Sapp and Bienkowska-Haba, 2009; DiGiuseppe et al., 2017). Additional binding to laminin 5 may enhance efficient binding to HS and/or collagen fibers, bringing viruses within close vicinity to cells interacting with the matrix. HS binding induces a conformational change in the viral capsid proteins L1 and L2 allowing HPV-16 viruses that are attached to HSPG in the ECM to detach and bind to secondary HSPG binding sites on the cell membrane (Sapp and Bienkowska-Haba, 2009). The interaction between HPV and HS is predominantly dependent on charge distribution and not sequence specific (Knappe et al., 2007). This allows the virus to utilize similar PG/GAGs for initial attachment and subsequent cell invasion independent of tissue source. Influenza and coronaviruses utilize highly conserved sialoglycan binding sites for cell invasion and it has been suggested for certain coronaviruses that HSPG attachment preceded sialic acid receptor binding (Owczarek et al., 2018; Tortorici et al., 2019).

**TABLE 2:** Pathogens exploiting the ECM for invasion.

Pathogen	ECM molecule(s) bound	Infection strategy
*S. aureus*	Laminin, collagen I, IV, VI, elastin, FN Singh et al., 2012	Tissue invasion and infection
*Candida albicans*	Laminin, collagen I, IV, FN Singh et al., 2012	Tissue attachment and enrichment
HPV-11	Laminin 5 Culp et al., 2006	Exploits interaction of α6-integrin with laminin 5 for keratinocyte invasion
HPV-16	HSPG, laminin 5, heparan sulfate Sapp and Bienkowska-Haba, 2009; DiGiuseppe et al., 2017	Used for attachment to BM, interstitial matrix or cell membranes prior to infection, brings virus in close vicinity of target cell that interacts with matrix
Polyomavirus	Laminin 5, collagen IV at BM Singh et al., 2012	Large T-antigen on virus binds to BM proteins
Merkel cell polyomavirus	Sulfated GAGs i.e., heparan sulfate; Matrosovich et al., 2013; Becker et al., 2019	Binding used to attach to sialic acid receptors on fibroblasts for cell invasion
HSV family	HSPG on cell surface Hadigal et al., 2020	Used for initial attachment before invasion
Coronavirus	HSPG Lang et al., 2011; Milewska et al., 2014; Owczarek et al., 2018	Enrichment of viruses around cells to enhance infection

*The list shown is not exhaustive. S. aureus, Staphylococcus aureus; FN, fibronectin; HPV, human papillomavirus; HSV, herpes simplex virus; HSPG, heparan sulfate proteoglycan; GAG, galactosaminoglycan/glycosaminoglycan; BM, basement membrane.*

Unlike bacteria, viruses do not encode their own ECM-remodeling metalloproteinases, but are still able to modify the host ECM indirectly (Alfano et al., 2016). Some viruses have been shown to upregulate cell adhesion molecules, such as the matrix ligand ICAM-1 (Parapoxviruses; Schneider et al., 2019), while others directly modify the cellular cytoskeleton (Smith et al., 2008; Bienkowska-Haba and Sapp, 2011; Nikolic et al., 2011; Stavolone and Lionetti, 2017). Both strategies can potentially change the force load on the matrix, either by upregulating cell binding to the matrix, inducing filopodia, or by increasing or decreasing pulling forces exerted by the cells through F-actin modifications (Taylor et al., 2011; Table 3). Proinflammatory cytokines are secreted *en masse* by activated immune cells upon virus infection resulting in increased activation of MMPs and altered ECM deposition (Han et al., 2001). As such, viruses can indirectly induce ECM remodeling by increasing collagenase expression in tissues. These posttranslational modifications of ECM components resemble a very basic mechanical way to escape the immune system by destroying the scaffold for immune cells to hold on to and also to increase tissue invasion by removing physical barriers for viral diffusion. The E2 protein of papillomavirus, for instance, induces MMP-9 expression and results in collagen degradation (Behren et al., 2005). Talmi-Frank et al. (2016) showed in a mouse model that influenza infection resulted in upregulation of MT1-MMP expression in myeloid CD11b+CD45+ cells causing collateral tissue damage in the lung. Interestingly, infected mice rather died by uncontrolled host immune responses instead of the virus itself. Only the combined treatment of inflammation and the control of viral load could rescue infected mice, highlighting the importance of balancing immune response and tissue integrity for survival. This has also been shown for coronavirus infections, where an unbiased mathematical modeling approach identified that wound repair and ECM remodeling pathways determined lethal versus sublethal disease outcome (Gralinski et al., 2013). SARS patients suffered severe lung injury and the urokinase pathway proved to be an essential regulator of disease outcome. High SARS-CoV-2 loads resulted in lung wounding and tissue thickening with increased keratin, fibronectin and plasminogen levels as a result of acute lung injury through tissue destruction (Gralinski et al., 2013). ECM degradation can result in anoikis in healthy tissues. Oncoviruses can affect tissue cell responses to changes in ECM attachment. Papillomavirus, Hepatitis virus and Epstein–Barr-virus have all been shown to promote cell resistance to anoikis, thereby allowing cancer cell invasion and metastasis (Kakavandi et al., 2018). In contrast, rhinovirus infection of airway smooth muscle cells has been shown to increase ECM deposition, in particular fibronectin, perlecan and collagen type IV, via activation of TLR3 and TLR7/8 (Kuo et al., 2011). Also Hepatitis viruses, which infect liver cells, can upregulate collagen deposition and cause liver fibrosis in the absence of acute inflammation (Ye et al., 2015; Stavolone and Lionetti, 2017) whereas other viruses like Zika decrease collagen production (Aguiar et al., 2020). Enhanced or reduced attachment of cells to the matrix can also have an enormous effect on the matrix force load due to in- or decreased cell-induced contraction. Thereby viruses can also indirectly modify physical parameters of the matrix and influence immune cell migration to infection sites.

**TABLE 3:** Viruses can modify the quantity and quality of the ECM directly and indirectly.

Virus	Involved molecules	Potential effect on matrix
Rhinovirus	TLR3, TLR7/8 Kuo et al., 2011	Increase in ECM deposition
Parapoxvirus	ICAM Schneider et al., 2019	Increased ICAM expression can enhance matrix interaction and increase load on matrix
Papillomavirus, HIV, SARS-CoV2	host actin cytoskeleton Smith et al., 2008; Bienkowska-Haba and Sapp, 2011; Nikolic et al., 2011; Bouhaddou et al., 2020	Changes in cell shape, cell protrusions or cellular tension can alter pulling forces on matrix
Papillomavirus	E2 protein, MMP-9 Behren et al., 2005	MMP-9 induction induces collagen degradation
Influenzavirus	MT1-MMP Talmi-Frank et al., 2016	MMP upregulation in immune cell can damage ECM
Coronavirus	Urokinase Gralinski et al., 2013	ECM degradation in lung can cause wounding and tissue thickening
HBV	Collagen type I, type III, TIMP, TGF-βYe et al., 2015	Upregulated collagen deposition and tissue fibrosis
Zika virus	TGF-β, collagen type I, LOX Aguiar et al., 2020	Reduced matrix deposition
HBV	Viral oncoprotein HBx Tse et al., 2018	Stabilization of HIF-1 can upregulate LOX
Merkel cell polyomavirus	ADAM 10 and 17 Nwogu et al., 2018	Increase in cellular sheddases remove cell adhesion receptors and can reduce force load on matrix

*Shown are examples of virus ECM modifications. The list of viruses and molecules is not exhaustive. ICAM, intercellular adhesion molecule; HIV, human immunodeficiency virus; MMP, matrix metalloproteinase; HBV, hepatitis B virus; TGF-β, transforming growth factor beta; HIF-1, hypoxia inducible factor 1; LOX, lysyl oxidase; ADAM, a disintegrin and metalloproteinase.*

**FIGURE 6:**
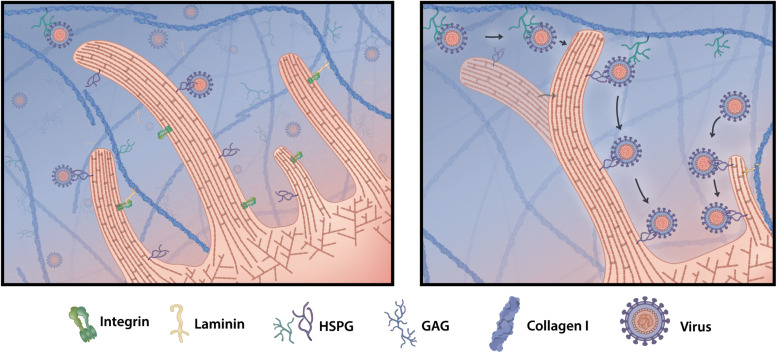
Filopodia grab virus particles off the matrix. Viruses that enter tissues stick to ECM proteins, such as HSPG, or freely diffuse in the interstitial fluid. Upon cell infection some viruses modify the cellular cytoskeleton and are able to induce cell protrusions called filopodia. When filopodia explore and interact with components of the ECM, they encounter freely floating or HSPG-bound virus particles and pick them off the matrix. Viruses hop from matrix HSPG onto secondary cell membrane HSPG and can surf along the filopodia shaft toward the cell body, where they invade and infect the target cell.

Further, viruses can influence qualitative changes to the ECM, such as crosslinking or tension load. Many changes to the physical ECM properties are initiated through oxidative stress and tissue inflammation caused by viral infections. Hepatitis B virus (HBV) encodes the viral onco-protein transactivator protein X (HBx), which interacts with and stabilizes HIF-1, a hypoxia-induced transcription factor (Tse et al., 2018). Thereby viruses can indirectly upregulate LOX expression resulting in LOX-dependent collagen crosslinking and subsequent stiffening of the matrix. Merkel cell polyomavirus induces carcinoma in mostly immunocompromised patients and disease progression has been associated with aging and prolonged UV exposure, suggesting a potential link between matrix stiffening and oncogenic progression of infection (Becker et al., 2019). Immune system responses to viral infections often cause tissue inflammation. This is frequently associated with changes to the ECM and tissue stiffening, which may enhance interactions of tissue-resident cells with the matrix. Effects on the host cytoskeleton through stiffened environments can for instance alter filopodia formation (Bienkowska-Haba and Sapp, 2011). Even slight stiffening in the matrix can have enormous effects on filopodia number, length and activity (Pfisterer et al., 2020). Recent studies show that certain viruses have adapted to be taken up by filopodia and thereby directly exploit and benefit from immune system-induced matrix modifications for increasing their cell–cell contact independent infection rate (Figure 6). Filopodia sense the topology of the matrix and can thereby brush off virus particles that are concentrated in the matrix and around cells. Recently, it was found that SARS-CoV-2 induces the formation of long branched filopodia in infected cells and this may be a potential strategy to efficiently invade cells (Bouhaddou et al., 2020). This potential is not limited to coronavirus and other viruses can also modify the host actin machinery, suggesting a conserved mechanism for efficient tissue infection (Taylor et al., 2011). For instance, HPV-31, which mainly infects basal keratinocytes, is able to actively change the host cytoskeleton and induce filopodia formation (Smith et al., 2008). HPV has also been shown to hop onto filopodia and surf along the filopodium shaft to the cell body in an actin- and myosin-dependent mechanism, before receptor binding and endocytosis (Lehmann et al., 2005; Smith et al., 2008). Virus-induced filopodia formation further increases uptake of freely diffusive virus particles from the ECM and may represent a yet less explored strategy for viral invasion into tissues.

## Concluding Remarks

Exciting new studies are highlighting the potential impact of ECM in maintaining health and how diseases impact the structure and composition of the ECM. However, knowledge in this field is still limited and further studies will be needed to investigate the role of the ECM in health and disease. As one of the most accessible peripheral organs, the skin represents an ideal model system for investigating ECM changes in inflammation, infections and its associated effects on tissue resident cells, such as immune cells (Tajpara et al., 2019). Novel 3D models and engineered biomaterials enable researchers to investigate biological processes in a physiological *in vitro* setting using defined parameter and further allow mathematical modeling approaches to answer questions (Chaudhuri et al., 2020). Many studies helped decipher the role of the ECM in cancer cell migration and recent reports suggest that modifying the matrix could be beneficial in the treatment of cancer and prevention of metastasis (Bonnans et al., 2014; Shen et al., 2020; Zhang and Reinhart-King, 2020). Inflammatory skin diseases and deregulated wound healing processes can result in aberrant deposition and remodeling of ECM components and cause fibrosis. Further studies are needed to clarify, whether enzymatic and non-enzymatic matrix modifications play a role in the initiation or sustainment of inflammatory skin diseases. The identification of potential links between hyperproliferative skin diseases, such as psoriasis, and matrix modifications could provide new anchor points for therapeutic interventions. Viral and bacterial infections can also deregulate tissue homeostasis either directly or indirectly. Interestingly, these processes seem to be independent of the target tissue and are further highly conserved between different viruses. Human skin could serve as an easily accessible model tissue to study viral invasion with a focus on tissue matrix composition, direct matrix modification or indirect changes to the force load on the matrix through modification of the cytoskeleton in tissue resident cells. As such, inflammation caused by viral infection or chronic inflammatory diseases could also greatly benefit from mechanomedicine. However, little research has been done beyond cancer research so far, leaving many open questions to be answered in future studies.

## Author Contributions

KP and WW wrote the manuscript. LS proofread and edited the manuscript. DS prepared the figures with help from KP. All authors read and approved the manuscript.

## Conflict of Interest

The authors declare that the research was conducted in the absence of any commercial or financial relationships that could be construed as a potential conflict of interest.
